# Role of miR-96/EVI1/miR-449a Axis in the Nasopharyngeal Carcinoma Cell Migration and Tumor Sphere Formation

**DOI:** 10.3390/ijms21155495

**Published:** 2020-07-31

**Authors:** Lai-Sheung Chan, Hong-Lok Lung, Roger Kai-Cheong Ngan, Anne Wing-Mui Lee, Sai Wah Tsao, Kwok-Wai Lo, Michael Kahn, Maria Li Lung, Rotraud Wieser, Nai-Ki Mak

**Affiliations:** 1 Department of Biology, Hong Kong Baptist University, Kowloon Tong, Hong Kong, China; ivy_chan@hkbu.edu.hk; 2 Department of Chemistry, Hong Kong Baptist University, Kowloon Tong, Hong Kong, China; hllung2@hkbu.edu.hk; 3 Department of Clinical Oncology, University of Hong Kong, Pokfulam, Hong Kong, China; rkcngan@hku.hk (R.K.-C.N.); awmlee@hku.hk; (A.W.-M.L.); mlilung@hku.hk (M.L.L.); 4 Center for Nasopharyngeal Carcinoma Research, University of Hong Kong, Pokfulam, Hong Kong, China; gswtsao@hku.hk; 5 Department of Anatomy, University of Hong Kong, Pokfulam, Hong Kong, China; 6 Department of Anatomical and Cellular Pathology and State Key Laboratory in Oncology in South China, The Chinese University of Hong Kong, Central Ave, Hong Kong, China; kwlo@cuhk.edu.hk; 7 Department of Molecular Medicine, Beckman Research Institute at City of Hope, Duarte, CA 91010-3000, USA; mkahn@coh.org; 8 Division of Oncology, Department of Medicine I, Medical University of Vienna, 1090 Vienna, Austria; rotraud.wieser@meduniwien.ac.at

**Keywords:** nasopharyngeal carcinoma, EVI1, miR-96, miR-449a, ICG-001

## Abstract

The Wnt signaling pathway is one of the major signaling pathways used by cancer stem cells (CSC). Ecotropic Viral Integration Site 1 (EVI1) has recently been shown to regulate oncogenic development of tumor cells by interacting with multiple signaling pathways, including the Wnt signaling. In the present study, we found that the Wnt modulator ICG-001 could inhibit the expression of EVI1 in nasopharyngeal carcinoma (NPC) cells. Results from loss-of-function and gain-of-function studies revealed that EVI1 expression positively regulated both NPC cell migration and growth of CSC-enriched tumor spheres. Subsequent studies indicated ICG-001 inhibited EVI1 expression via upregulated expression of miR-96. Results from EVI1 3′UTR luciferase reporter assay confirmed that EVI1 is a direct target of miR-96. Further mechanistic studies revealed that ICG-001, overexpression of miR-96, or knockdown of EVI1 expression could restore the expression of miR-449a. The suppressive effect of miR-449a on the cell migration and tumor sphere formation was confirmed in NPC cells. Taken together, the miR-96/EVI1/miR-449a axis is a novel pathway involved in ICG-001-mediated inhibition of NPC cell migration and growth of the tumor spheres.

## 1. Introduction

Nasopharyngeal carcinoma (NPC), a type of epithelial malignancy in the region of the nasopharynx, has a high frequency of occurrence in southern China. A previous large-scale genome-wide association study in 1583 NPC patients showed that *Ecotropic Viral Integration Site 1 (EVI1) and Myelodysplastic Syndrome 1* (*MDS1-EVI1*) is one of the susceptibility loci in NPC [1,2]. Recently, high EVI1 expression was found to correlate with not only the tumor size and distant metastasis, but also with the shorter overall survival of the NPC patients [3]. The protein encoded by *EVI1* is a zinc finger transcriptional regulator involved in the regulation of cell proliferation and differentiation [4]. EVI1 has been shown to be expressed in various developing tissues, such as the heart, respiratory system and nasal cavity [5]. EVI1 is also an important transcription factor involved in the regulation of hematopoietic stem cell growth [6,7]. It has been shown that dysregulated expression of EVI1 is involved in the oncogenic development of leukemia [8]. Overexpression of EVI1 is frequently associated with poor response to chemotherapy in myeloid leukemia [9,10,11]. The role of EVI1 in the oncogenic development of epithelial cancers has also been reported recently. In colon cancer, the expression of EVI1 was found to be upregulated in 53% of human colorectal cancer samples and all of the examined colon adenoma samples [12]. In hepatocellular carcinoma, a high expression level of EVI1 was found to correlate with a larger tumor size [13]. However, the contribution of EVI1 to the oncogenic development of NPC has not been fully studied.

MicroRNAs (miRNAs) are a group of small non-protein-encoding regulatory RNAs. The miRNAs mainly function as negative regulators to inhibit the process of protein translation. It is now clear that abnormal expression of miRNAs with oncogenic or tumor-suppressive function is associated with cancer development [14,15]. In NPC, aberrant expression of miRNAs has been reported [16,17]. Among all the aberrantly expressed miRNAs, miR-449a was found to be downregulated in the NPC tissues at all stages (stages I to IV and metastasis) [18]. However, the regulation and functional role of miR-449a has not been previously examined in NPC.

The highly developmentally conserved Wnt/β-catenin signaling pathway is frequently dysregulated in tumor cells. Upon receiving activation signals, β-catenin may interact with the coactivator CREB-binding protein (CBP) for the initiation of self-renewal/cell proliferation or interact with another highly homologous coactivator p300 for the initiation of the cell differentiation program [19,20]. A recent study indicated that EVI1 may form an interaction network with several major cellular pathways, including the Wnt signaling pathway [21]. The relationship between EVI1 and Wnt/β-catenin signaling has recently been described in NPC. EVI1 was found to regulate the cancer stem cell (CSC)-associated properties of NPC cells via binding to the β-catenin promoter and subsequent activation and expression of β-catenin and the Wnt/β-catenin downstream target AXIN2 [3]. However, the regulation of expression and also the biological actions of EVI1 in NPC cells are still unknown. We have previously demonstrated that the small specific molecule CBP/β-catenin antagonist ICG-001 can inhibit the migration and growth of CSC-enriched NPC tumor spheres via the miRNA-145/SOX2 (SRY-Box Transcription Factor 2) axis [22] and miR-150/CD44 axis [23]. In the present study, we further examined the underlying antitumor mechanisms of ICG-001 in NPC cells. We found that the miR-96/EVI1/miR-449a axis is involved in the inhibition of the growth and migration of NPC cells.

## 2. Results

### 2.1. ICG-001 Reduces the Protein Expression of EVI1 in C666-1 Cells

**Figure 1:**
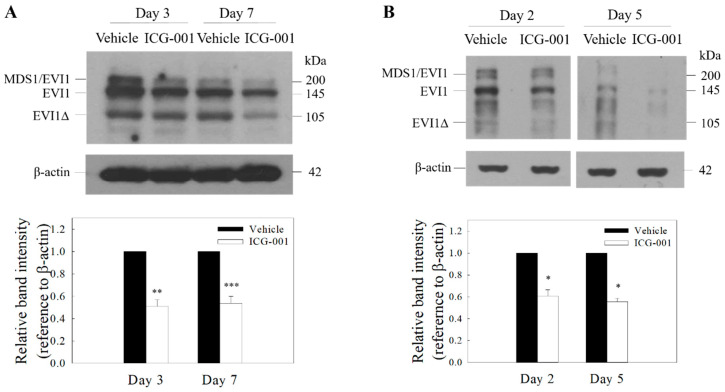
ICG-001 reduces the expression of Ecotropic Viral Integration Site 1 (EVI1) in EBV-positive nasopharyngeal carcinoma (NPC) cells. Western blot analysis of EVI1 in ICG-001-treated C666-1 (**A**) and C17 cells (**B**). The cells were treated with ICG-001 (10 µM) or an appropriately diluted DMSO (vehicle control). Representative images are presented. Signal intensities were determined by quantitative densitometry and expressed as fold change of EVI1 normalized to β-actin. Values are presented as mean ± SD of at least four independent experiments. * *p* < 0.05, ** *p* < 0.01, *** *p* < 0.001 compared with vehicle control.

Recent studies by Lu and co-workers showed that high EVI1 expression is highly correlated with certain clinicopathological characteristics such as tumor size, lymph node metastasis, distant metastasis and an advanced clinical stage in NPC [3]. EVI1 was also found to activate the Wnt/β-catenin signal pathway and promote cancer stem cell features [3]. In the present study, we sought to determine whether pharmacological intervention in the Wnt/β-catenin signal pathway with a selective CBP/β-catenin antagonist ICG-001 would have an effect on the expression of EVI1. The MDS1-EVI1 complex is known to be transcribed into several alternative mRNA variants. Results from Western blotting analysis showed that MSD1-EVI1, EVI1, and the smaller size EVI1∆ protein could be detected in both Epstein-Barr virus (EBV)-positive c666-1 and C17 NPC cell lines (Figure 1, full blot images were shown in Supplementary File). ICG-001 could significantly reduce the protein expression of EVI1 from day 2 to day 7 after the treatment.

### 2.2. Effect of EVI1 on the Migration and Growth of NPC Tumor Spheres

**Figure 2:**
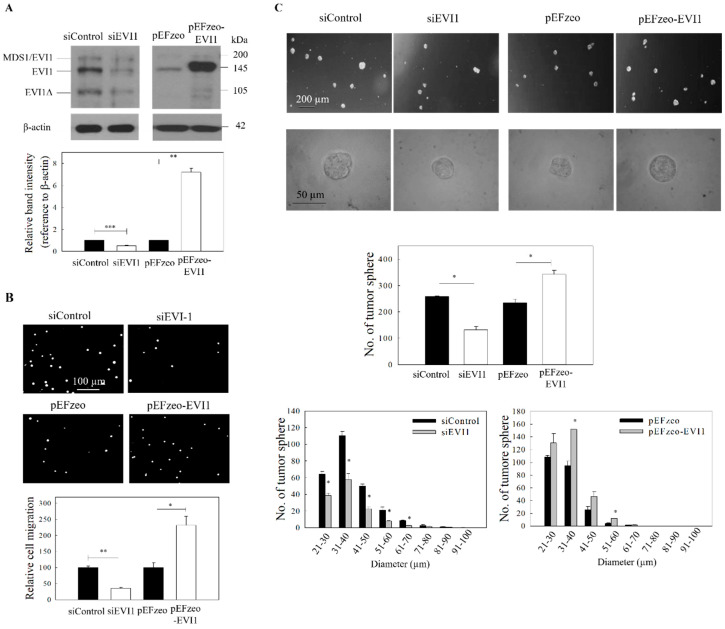
ICG-001 inhibits the migration and tumor sphere formation of C666-1 cells. Cells were transfected with either EVI1 siRNA pool (siEVI1) or expression plasmid (pEFzeo-EVI1). Negative control siRNA (siControl) or pEFzeo vector without the target insert were used as controls. (**A**) The transfection efficiency was assessed by Western blotting analysis. Representative images are presented in the upper panel, and signal intensities were determined by quantitative densitometry and expressed as fold change of EVI1 normalized to β-actin in the lower panel. (**B**) Migration of the transfected cells was assessed by transwell migration assay. Upper panel: images of the migrated cells. Lower panel: quantitative measurement of the migrated cells. (**C**) Tumor sphere formation assay. The transfected cells were harvested and subjected to tumor spheroid formation assay. Representative bright-field images (upper panel), total number (middle panel) and size distribution of tumor spheres (lower panel) are presented. At least three independent experiments were carried out. * *p* < 0.05; ** *p* < 0.01; *** *p* < 0.001 compared with the corresponding control.

The functional activity of EVI1 in NPC cells was subsequently examined by loss-of-function (using EVI1 siRNA) and gain-of-function (overexpression using pEFzeo-EVI1 vector) studies. The effect of siRNA silencing and EVI1 overexpression was first confirmed by Western blot. Results in Figure 2A indicated that siRNA and pEFzeo-EVI1 could reduce and overexpress, respectively, in C666-1 NPC cells (full blot images were shown in Supplementary File). In Figure 2B, the migration of NPC cells was significantly reduced in cells after transfection with EVI1 siRNA. In contrast, overexpression of EVI1 resulted in a significant increase in cell migration. A similar approach was also used to examine the impact of EVI1 expression on the formation of tumor spheres. Results from the spheroid size profile analysis (Figure 2C) showed that both the size and number of NPC tumor spheres were reduced in cells after EVI1 siRNA treatment. In contrast, the size and number of NPC tumor spheres increased after overexpression of pEFzeo-EVI1.

### 2.3. ICG-001-Mediated Downregulation of EVI1 Is Regulated by miR-96

**Figure 3:**
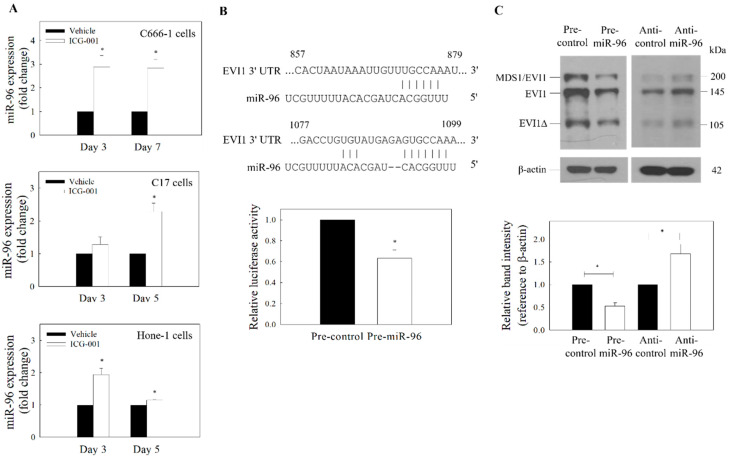
Expression of miR-96 and EVI1. (**A**) qRT-PCR analysis of ICG-001-induced expression of miR-96. ICG-001 upregulates the expression of miR-96 in the EBV-positive (C666-1 and C17) and EBV-negative (Hone-1) cells. (**B**) Putative binding site of miR-96 on EVI1 mRNA 3′-UTR was predicted using TargetScan (upper panel). The EVI1 3′UTR luciferase reporter assay (lower panel) was performed in C666-1 cells. (**C**) Effect of miR-96 on the expression of EVI1. C666-1 cells were transfected with pre-miR-96 or anti-miR-96. Upper panel: Protein blot. Lower panel: Quantitative analysis of EVI1 expression from the protein blot. Values are presented as the mean ± SD of at least three independent experiments. * *p* < 0.05 compared with the corresponding control.

**Figure 4:**
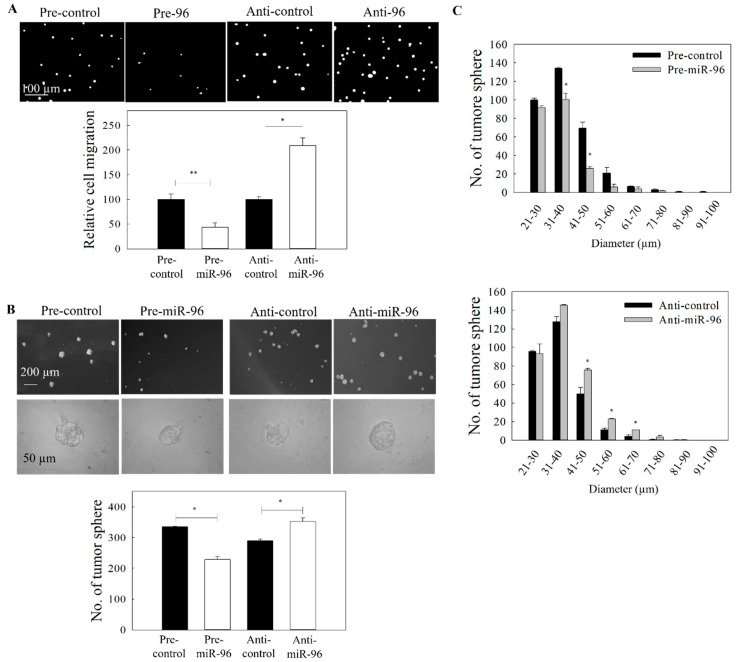
Effect of miR-96 expression on the migration and tumor sphere formation. C666-1 cells were transfected with either pre-miR-96 or anti-miR-96. (**A**) The migration of the transfected cells was assessed by the transwell migration assay. Upper panel: images of the migrated cells. Lower panel: quantitative analysis of the migrated cells. (**B**) Tumor sphere formation assay. Upper panel: images of tumor spheres in culture well of ultralow attachment plate. Lower panel: quantitative analysis of spheroid number. (**C**) Size distribution of tumor spheres from (**B)**. At least three independent experiments were carried out. * *p* < 0.05; ** *p* < 0.01 compared with the corresponding control.

miR-96 has previously been implicated in EVI1-mediated growth control of pancreatic cancer [24]. In NPC, the expression of miR-96 had been reported to be downregulated in CSC-enriched tumor spheres [25]. In our preliminary bioinformatic analysis of the 3′UTR mRNA from TargetScan, putative binding sites of miR-96 were found in the 3′UTR of EVI1 mRNA. We first examined whether ICG-001 could restore the expression of miR-96 in NPC cells. Results from Figure 3A show that ICG-001 could upregulate miR-96 from day 3 to day 7 after treatment. According to the TargetScan prediction, there are two putative miR-96 target sites (857–879 and 1077–1099) at the 3′UTR. A vector containing these sequences was then used in an EVI1 luciferase reporter assay. Results from Figure 3B clearly show that miR-96 could inhibit the 3′-UTR activity, indicating that EVI1 is a target of miR-96. The impact of miR-96 expression on EVI1 was further examined by transfection of NPC cells with precursor miR-96 (pre-miR-96). Results in Figure 3C show that the protein expression of EVI1 was reduced (full blot images were shown in Supplementary file). In the control experiments, transfection of the cells with miR-96 inhibitor (anti-miR-96) could increase the expression of EVI1. To further confirm that the miR-96/EVI1 axis is involved in NPC cell migration and growth of tumor spheres, cells were transfected with pre-miR-96 or anti-miR-96. Results from the gain-of-function (pre-miR-96 transfection) studies indicate that overexpression of miR-96 could significantly inhibit both the tumor cell migration (Figure 4A) and growth of tumor spheres (Figure 4B,C). In the control experiment, anti-miR-96 could increase the migration and growth of tumor spheres. Transfection efficiency of the miR-96 precursor and miR-96 inhibitor are shown in Supplementary Figure S1. Taken together, ICG-001 could inhibit the formation of tumor spheres and migration of NPC cells, at least partially via the miR-96/EVI1 axis.

### 2.4. Effects of EVI1 Downregulation on the Expression of miR-449a

**Figure 5:**
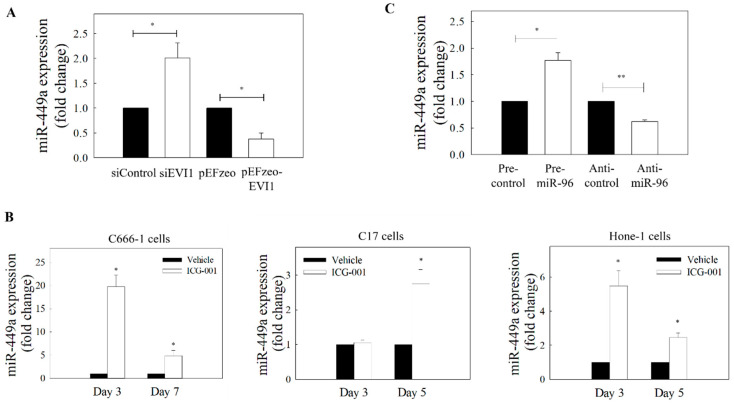
EVI1 downregulates miR-449a in NPC cells. (**A**) Expression of miR-449a in C666-1 cells transfected with EVI1 siRNA or pEFzeo-EVI1 plasmid. (**B**) Expression levels of miR-449a in ICG-001-treated EBV-positive (C666-1 and C17) and EBV–negative (Hone-1) NPC cells. (**C**) Effect of miR-96 on the expression of miR-449a in C666-1 cells. At least three individual experiments were performed. * *p* < 0.05, ** *p* < 0.01 compared with the corresponding control.

Increasing evidence has demonstrated that miR-449a is an important tumor suppressor [26]. A previous mechanistic study showed that miR-449a is a direct target of the transcriptional repressor EVI1 [27]. Two previous independent miRNA profile analyses on NPC tissues showed that the expression of miR-449a was downregulated when compared with normal nasopharyngeal epithelial samples [18] or adjacent normal nasopharynx tissues [16]. Since EVI1 has been reported to be overexpressed and associated with pathogenesis of NPC [3], we hypothesized that knockdown of EVI1 expression might increase the expression of miR-449a in NPC cells. Results in Figure 5A showed that knockdown of EVI1 could increase the expression level of miR-449a in NPC cells. In contrast, the expression of miR-449a could be reduced after EVI1 overexpression. In addition, treatment of NPC cells with ICG-001 also restored the expression of miR-449a (Figure 5B). Since miR-96 was found to be the upstream regulator of EVI1, we hypothesized that the expression of miR-449a should be affected by miR-96. Figure 5C clearly shows that transfection of cells with pre-miR-96 could significantly increase the expression of miR-449a, and the expression was reduced by anti-miR-96.

### 2.5. Functional Activity of miR-449a

**Figure 6:**
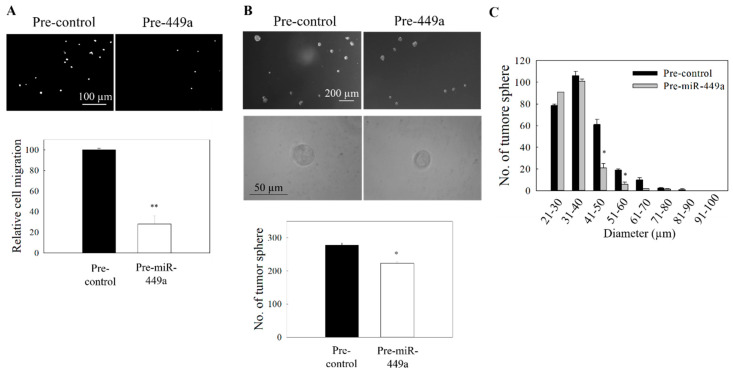
Effect of miR-449a on the migration and tumor sphere formation. C666-1 cells were transfected with pre-miR-449a. (**A**) Transwell migration of transfected cells. Upper panel: images of the migrated cells. Lower panel: quantitative analysis of the cells migration. (**B**) Tumor sphere formation assay. Upper panel: images of tumor spheres in culture well of ultralow attachment plate. Lower panel: quantitative analysis of spheroid number in pre-miR449a transfected cells. (**C**) Size distribution profile of tumor spheres. At least three independent experiments were carried out. * *p* < 0.05; ** *p* < 0.01 compared the control group.

**Figure 7:**
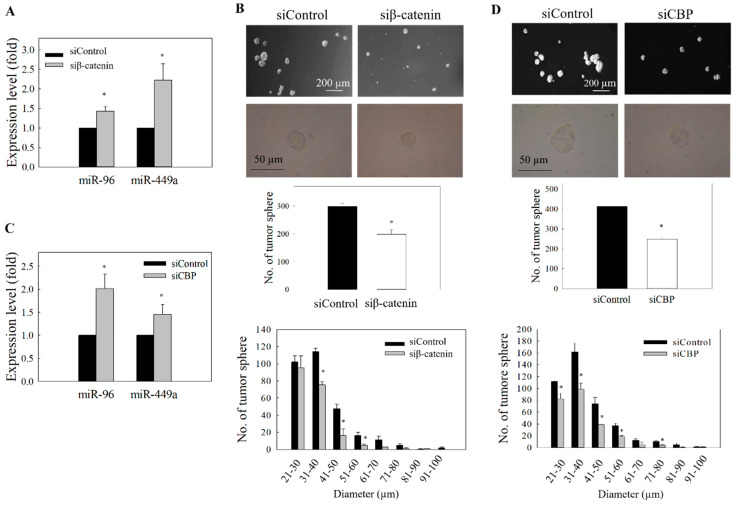
Effect of β-catenin and CBP knockdown on the expression of miR-96 and miR-449a. (**A**) C666-1 cells were transfected with β-catenin siRNA. Expression of miR-96 and miR-449a was determined by qRT-PCR. (**B**) Quantitative determination of tumor sphere formation and size profile after transfection of β-catenin siRNA. (**C**) Cells were transfected with CBP siRNA, and the expression of miR-96 and miR-449a was determined by qRT-PCR. (**D**) Quantitative determination of tumor sphere formation and size profile after transfection with CBP siRNA. At least three independent experiments were carried out. * *p* < 0.05 compared with corresponding control.

In the subsequent functional studies, the impact of miR-449a overexpression on the NPC cell migration and also the formation of tumor spheres was examined. MiR-449a was overexpressed by transfecting the NPC cells with pre-miR-449a, and the transfection efficiency is shown in Supplementary Figure S2. Results in Figure 6A show that the migration of NPC cells was significantly reduced after pre-miR-449a transfection. Results from the spheroid size profiling study also showed that the size of tumor spheres was reduced (Figure 6B,C). Finally, to reconfirm that both miR-96 and miR-449a are under the control of Wnt/β-catenin signaling, we examined whether knockdown of β-catenin or the co-activator CBP could phenocopy the effect of ICG-001-mediated restoration of miR-96 and miR-449a in NPC cells. Results show that treatment of cells with siRNA for β-catenin (Figure 7A) or siRNA for CBP (Figure 7B) resulted in the upregulation of expression of miR-96 and miR-449a, and the effect was accompanied by the inhibition of tumor cell migration and the formation of tumor spheres (Figure 7C,D). Taken together, the miR-96/EVI1/miR449a axis is involved in ICG-001-mediated inhibition of NPC cell migration and spheroid formation.

## 3. Discussion

Wnt/β-catenin is one of the major CSC-associated signaling pathways. In general, there are multiple mechanisms contributing to the dysregulation of the Wnt signaling in tumor cells. For example, loss-of-function mutation of Adenomatous Polyposis Coli (*APC*), gain-of-function mutation of β-catenin, overexpression of the Wnt receptor/ligands, and the reduced expression of the Wnt-negative regulators. In NPC, a previous genomic landscape study revealed that the mutation rate of NPC is relatively low, and significant mutation of APC or β-catenin was not observed [28]. However, both the host cell and EBV viral factor-mediated inhibition of expression or silencing of the Wnt-negative regulators appear to contribute to the dysregulation of Wnt signaling in NPC. These include (i) epigenetic silencing of some major negative Wnt regulators (e.g., methylation of WNT Inhibitory Factor 1 (*WIF-1*) and *APC* genes) [29,30]; (ii) phosphorylated inactivation of Glycogen Synthase Kinase 3 Beta (GSK3β) [31]; (iii) dysregulation of the expression of miRNA for the Wnt pathway [16,17]; (iv) targeting of the negative Wnt regulators (e.g., WIF1, APC, Nemo Like Kinase (NLK)) by the EBV-encoded miRNAs [32]; (v) activation of β-catenin via epigenetic silencing of the receptor Receptor Tyrosine Kinase Like Orphan Receptor 2 (ROR2) (a negative regulator) in the non-canonical Wnt pathway [33] and (vi) the high level of expression of the β-catenin coactivator CBP [34,35]. In view of the widespread dysregulated expression of the Wnt-regulated signaling molecules in NPC, we hypothesized that selective pharmacological intervention at the distal end of the Wnt/β-catenin signaling pathway, namely the interaction between β-catenin and the co-activator CBP by the CBP antagonist ICG-001 [19,20], might be a novel strategy to control the growth and migration of NPC cells. We are especially interested in the role of cellular miRNA in ICG-001-mediated anti-tumor activity.

The miRNAs are known to regulate a wide range of biological process. These include self-renewal, cell growth and differentiation, and cell migration [36,37]. In NPC, dysregulation of miRNA for the Wnt pathway has been well documented [16,17]. Especially the expression of miR-449a was found to be downregulated from stages I to IV and also metastatic NPC [18]. Downregulated expression of miR-96 was also observed in the CSC-enriched NPC tumor spheres [25]. The observation of downregulated expression of miR-96 [25] and high expression of EVI1 in NPC [3] is worthy of discussion. It has previously been demonstrated in pancreatic cancer that miR-96 could suppress the expression of EVI1 via the binding with EVI1 3′UTR, and EVI1 could transcriptionally suppress the expression of miR-96 through binding with the EVI1 potential binding site around miR-96 (24). The balance between EVI1 and miR-96 and the EVI1/miR-96/KRAS axis appears to play an important role in regulating the growth of pancreatic tumor cells. In NPC, we confirmed that miR-96 could downregulate the expression of EVI1 (Figure 3). Similar reciprocal regulation between miR-96 and EVI1, namely the effect of EVI1 on the expression of miR-96, was also observed in NPC (Supplementary Figure S3). In the present study, we further demonstrate that this reciprocal regulation could be interrupted by increasing the expression of miR-96 and, hence, reducing the EVI1 expression with the CBP antagonist ICG-001.

In the subsequent functional study, we found that EVI1/miR-449a axis is involved in the regulation of spheroid growth and also the migration of NPC cells. Although the mechanism of miR-449a-mediated inhibition was not further examined in the present study, Li and co-workers had previously demonstrated that miR-449a could regulate glycolysis and inhibit the growth of NPC cells by targeting lactate dehydrogenase A (LDHA) [38]. Apart from LDHA, miR-449a had also been shown to inhibit the growth of endometrial cancer, osteosarcoma, breast cancer, glioma and non-small-cell lung cancer cells by targeting the steroid receptor coactivator [39], Enhancer Of Zeste 2 Polycomb Repressive Complex 2 (EZH2) [40], Pleomorphic adenoma gene like-2 [41], Notch Receptor 1 (Notch1) [42] and High Mobility Group Box 1 (HMGB1) [43], respectively. In NPC, EZH2 has been shown to be overexpressed in biopsy samples [44] and has been functionally implicated in the control of cell growth and cell migration [45,46]. We previously demonstrated that ICG-001 could downregulate the protein expression of EZH2 in NPC cells [22]. Taken together, the tumor-suppressive activity of miR-449a in NPC cells might be attributed to the inhibition of expression of multiple cell growth regulators, such as LDHA and EZH2.

**Figure 8:**
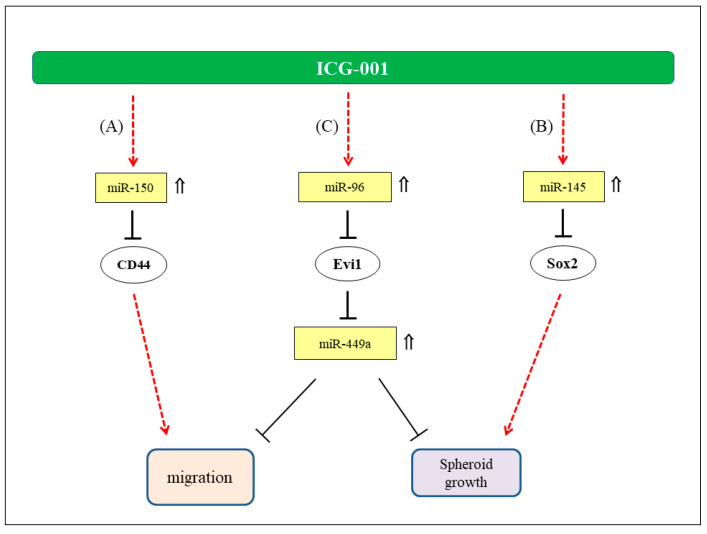
Hypothetical mode of anti-tumor action of ICG-001 in NPC. (**A**) Involvement of miR-150/CD44 axis in the migration of NPC cells (23). (**B**) Regulation of NPC tumor spheres growth via miR-145/SOX2 axis (22). (**C**) Regulation of NPC cell migration and growth of tumor spheres via miR-96/Evi1/miR-449s axis. ⇑: Upregulated expression in ICG-001-treated NPC cells.

Apart from the miR-449a, we have previously demonstrated that ICG-001 could also inhibit the growth of CSC-enriched NPC tumor spheroids via miR-145/SOX2 axis [22] and the NPC cell migration via miR-150/CD44 axis [23]. Figure 8 summarizes the hypothetical mode of action of ICG-001 on NPC cells. The implication of restoration of multiple miRNAs with anti-tumor activities in NPC cells is worthy of further discussion. Underexpression of miRNAs with tumor-suppressive properties is a general phenomenon in tumor cells [47]. For this reason, tumor-suppressive miRNAs are frequently considered as potential therapeutic agents in cancer treatment. Many tumor-suppressive miRNAs, including miR-449a and miR-145, have been implicated as the candidate therapeutic agents [26,48,49]. When compared with the use of a single species of tumor-suppressive miRNA, modulation of Wnt/β-catenin signaling and the subsequent increased expression of multiple tumor-suppressive miRNAs and, hence, inhibition of multiple growth controlling regulators may be a novel approach in the treatment of NPC.

The significance of ICG-001-mediated downregulation of EVI1 expression in NPC is worthy of further discussion. EVI1 is a well-known oncogenic protein in various cancers. Previous network analysis showed that EVI1 could interact with several signaling pathways including Wnt, TGF-β (Transforming Growth Factor Beta) and Ras (Rat sarcoma) [21]. Lu and co-workers found that EVI1 could promote the CSC properties of EBV-negative NPC cells via the Wnt/β-catenin signaling pathway [3]. However, the function of EVI1 in the EBV-positive NPC cell lines was not further examined. It is now clear that NPC is an EBV-associated malignancy. Study of the functional role of EVI1 in EBV-positive cells is very clinically relevant. In the present study, we established the interplay between miR-96, EVI1 and miR-449a in ICG-001-treated EBV-positive cells. In the mechanistic studies, we further demonstrated that EVI1 would regulate NPC cell migration and tumor sphere formation via miR-449a. This observation further supports the previous findings that miR-449a can function as a tumor suppressor to inhibit tumor cell migration, invasion and proliferation in other tumor cell types [39,40,41,50,51]. Apart from the regulation of miR-449a expression, previous global pathway analysis showed that EVI1 could bind to 78% of genes involved in the regulation of the Jak-Stat signaling [52]. In NPC, Zhang and co-workers demonstrated that interlukin 6/ signal transducer and activator of transcription 3 (IL6/Stat3) signaling may enhance the growth and tumorigenicity of EBV-positive tumor cells via the upregulation of the EBV LMP-1 viral protein [53]. Taken together, EVI1 can not only regulate the growth of NPC via the downregulation of expression of miR-449a but may also regulate the growth of NPC cells via the regulation of expression of Stat3 and LMP-1 viral protein. In conclusion, pharmacological intervention in the Wnt/β-catenin signaling with the CBP/β-catenin antagonist ICG-001 could inhibit tumor cell growth and migration, at least partially, via the miR-96/EVI1/miR-449a axis.

## 4. Materials and Methods

### 4.1. Cell Culture and Chemicals

The EBV-positive (C666-1 and C17) and EBV-negative (HONE-1) NPC cell lines were cultured as previously described [22,23]. C666-1 and HONE-1 cell lines were authenticated by and obtained from the Hong Kong NPC AoE Cell Line Repository. C17 cell line was obtained from Professor Pierre Busson, Université Paris-Sud, Paris, France. A stock solution of ICG-001 (20 mM) was prepared in DMSO.

### 4.2. Cell Transfection

NPC cells were transfected with siRNA or miRNA as previously described [22,23]. Briefly, NPC cells (3 × 10^5^) were cultured in fibronectin (6 µg/mL) pre-coated 35 mm culture plates overnight. Lipofectamine^®^ 2000 (Invitrogen, Thermo Fisher Scientific, Waltham, MA, USA) was used in all transient transfection experiments, according to the manufacturer’s protocols. In the siRNA knockdown experiment, cells were transfected with 100 nM ON-TARGETplus SMARTpool Human EVI1 siRNA (Dharmacon, Lafayette, CO, USA; Cat. No: ID L-006530-02-0005), 50 nM β-catenin siRNA (Ambion, Thermo Fisher Scientific, Waltham, MA, USA; Cat. No: AM16708; assay ID: 146154) or 50 nM CBP siRNA (Dharmacon; Cat. No: ID L-003486-00-0005) for 48 h. ON-TARGETplus siCONTROL Non-Targeting siRNA (Dharmacon; Cat. ID D-001810-01-20) or Silencer^®^ Negative Control #1 siRNA Ambion (Ambion; Cat. no. AM4611) was used in the corresponding control group.

For the transfection of precursor miRNA (pre-miRNA) or miRNA inhibitor (anti-miR), the cells were transfected with 50 nM pre-miR-96 (Ambion; P/N: AM17100; ID: PM10422), pre-miR-449a (Ambion; P/N: AM17100; ID: PM11127), or miR-96 inhibitor (anti-miR-96) (Ambion; P/N:AM17000; ID: AM10422) for 72 h. The miRNA precursor negative control (Pre-control, ID: AM17110) and miRNA inhibitor negative control (Anti-control, ID: AM17010) were obtained from Ambion. After transfection, the cells were collected for the subsequent analysis. Regarding the EVI1 plasmid transfection, 400 ng expression plasmid containing EVI1 cDNA (pEFzeo-EVI1) was transfected into NPC cells as described above. The same amount of the empty vector (pEFzeo) was used in the control experiment. The cells were then collected 24 h after transfection for the subsequent analysis.

### 4.3. 3’UTR miRNA Luciferase Reporter Assay

Bioinformatics software TargetScan Human (Release 7.1: June 2016) [54] was initially used for the prediction of potential targets of miR-96. To validate the target of miR-96, 10 ng of Evi1 3′UTR luciferase reporter (OriGene Technologies, Rockville, MD, USA; CAT#: SC214441) along with the 100 nM miR-96 mimic (Pre-miR-96) or miRNA mimic control (Pre-control) were transfected into C666-1 cells using Lipofectamine 2000 (Invitrogen) for 72 h. The luciferase activity was determined according to the manufacturer’s instruction. Briefly, the signal of red fluorescent protein (RFP) transcribed by the vector was captured under a fluorescent microscope. The total intensity of the RFP was then measured using MetaMorph software v1.5 (Leica Microsystems, Wetzlar, Germany) The cells were then lysed using passive reporter lysis buffer provided by Luciferase Assay System (Promega, Madison, WI, USA). Luciferase activities were measured using Luciferase Assay System with a microplate luminometer and normalized to the signals of RFP.

### 4.4. Tumor Sphere Formation Assay

CSC-enriched tumor sphere formation assay was performed as previously described [22]. Briefly, transfected C666-1 cells (2000 cells/well) were cultured in DMEM/F12 medium (Gibco) supplemented with 20 ng/mL EGF (Sigma-Aldrich, Merck KGaA, St. Louis, MO, USA), 20 ng/mL FGF (Cell Signaling Technology, Danvers, MA, USA) and 20 ng/mL IGF (Cell Signaling Technology) for 7 days. The size and images of the tumor spheres were captured and analyzed using Image J software v1.46r (National Institutes of Health, Bethesda, MD, USA).

### 4.5. Transwell Migration Assay

Cell migration assay was performed as described previously [23]. Briefly, transfected C666-1 cells were seeded onto 6.5 mm transwell inserts with an 8.0 μm pore polycarbonate membrane (Corning Incorporated, Corning, NY, USA). The cells were incubated in RPMI-1640 medium supplemented with 10% fetal bovine serum (FBS) and 1% Pen Strep (50 units/mL penicillin and 50 μg/mL streptomycin) for 24 h. The migrated cells at the bottom of the membrane were fixed in 4% paraformaldehyde, permeabilized in 0.2% Triton-X and stained with 4,6-Diamidino-2-phenylindole (DAPI) (Sigma-Aldrich). The migrated cells were then visualized and counted under a fluorescent microscope.

### 4.6. Western Blotting Analysis

Treated NPC cells were lysed in lysis buffer (250 mM Tris, pH 8; 1% NP-40 and 150 mM NaCl) containing 1% phosphatase inhibitors cocktail Set II (Calbiochem, Merck KGaA, Darmstadt, Germany) and 0.25% protease inhibitors cocktail (Sigma-Aldrich). The lysate was centrifuged at 14,000 rpm (10 min, 4 °C). Supernatant was collected, and the protein concentration was determined by DC Protein Assay Kit (Bio-Rad, Hercules, CA, USA). Protein denaturation was performed by boiling for 10 min in SDS (sodium dodecyl sulfate) sample buffer. Equal amounts of protein samples were then resolved in SDS-polyacrylamide gels and transferred to PVDF membranes (Millipore, Merck KGaA, Darmstadt, Germany). After blocking with 5% non-fat dry milk, the membranes were incubated with anti-EVI1 antibody (Cell Signaling Technology, Cat no: 2593). The membrane was then washed and incubated with HRP-conjugated secondary antibodies (Invitrogen, Cat. no.: 656120). Protein bands were detected using a chemiluminescent system (Lab Frontier, Anyang, Gyeonggi, Korea) and visualized on X-ray film. The β-actin was used as the internal control. Band intensities were analyzed by using ImageJ software v1.46r (National Institutes of Health, USA).

### 4.7. miRNA Expression Analysis

Total RNA was extracted using TRIzol Reagent (Ambion), according to manufacturer’s instruction. TaqMan MicroRNA Reverse Transcription Kit (Applied Biosystems, Thermo Fisher Scientific, Waltham, MA, USA) was used in reverse transcription. Real-time PCR was subsequently performed in StepOnePlus Real-time PCR System using TaqMan 2X Universal PCR Master Mix (No AmpErase UNG) (Applied Biosystems). Specific primer probes for the miR-96 (Assay ID: 000186) and miR-449a (Assay ID: 001030) were from TaqMan MicroRNA Assays (Applied Biosystems, Cat. no.: 4427975). Small nuclear RNA RNU6B (U6) (Assay ID: 001093) was used as internal control. The relative expression of target transcripts was calculated by 2^−ΔΔCt^ method.

### 4.8. Statistics

Data were presented as the mean ± standard deviation (SD) of at least three independent experiments. Statistical comparisons between two groups were determined by the Student t-test. Statistical significance was set as *p* < 0.05.

## Supplementary Materials

Supplementary materials can be found at https://www.mdpi.com/1422-0067/21/15/5495/s1.
