# Supplementary material for: Role of miR-96/EVI1/miR-449a Axis in the Nasopharyngeal Carcinoma Cell Migration and Tumor Sphere Formation
Source: Int J Mol Sci. 2020 Jul 31;21(15):5495. doi: 10.3390/ijms21155495 (PMC7432346; doi:10.3390/ijms21155495)

Original western blot image

Figure 1A

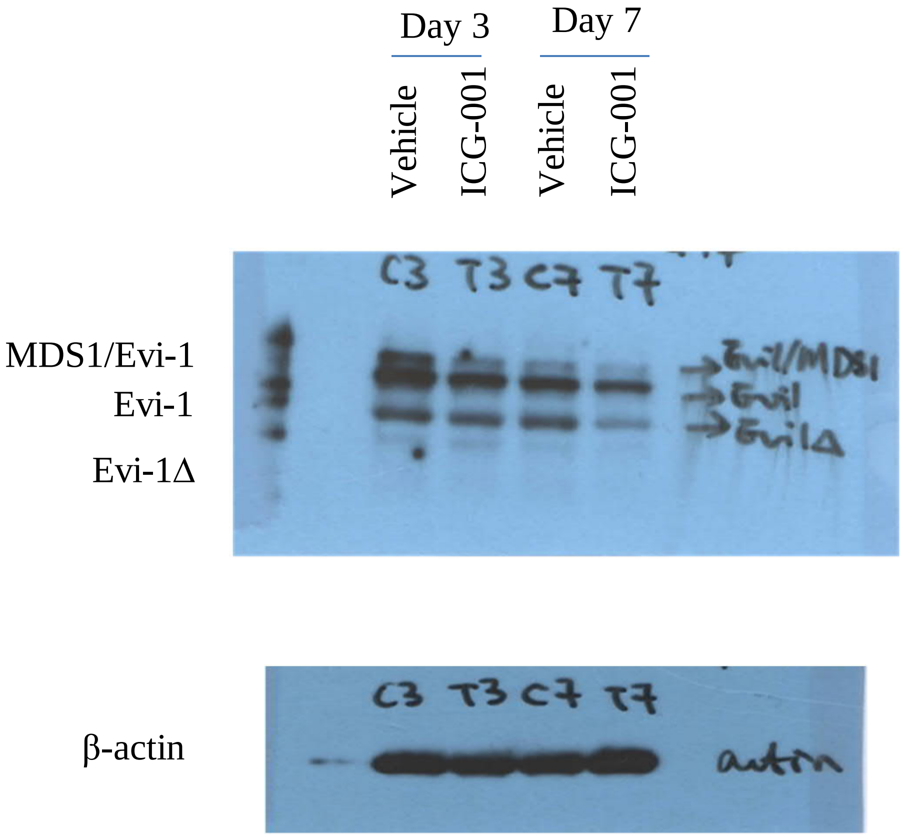

Figure 1B

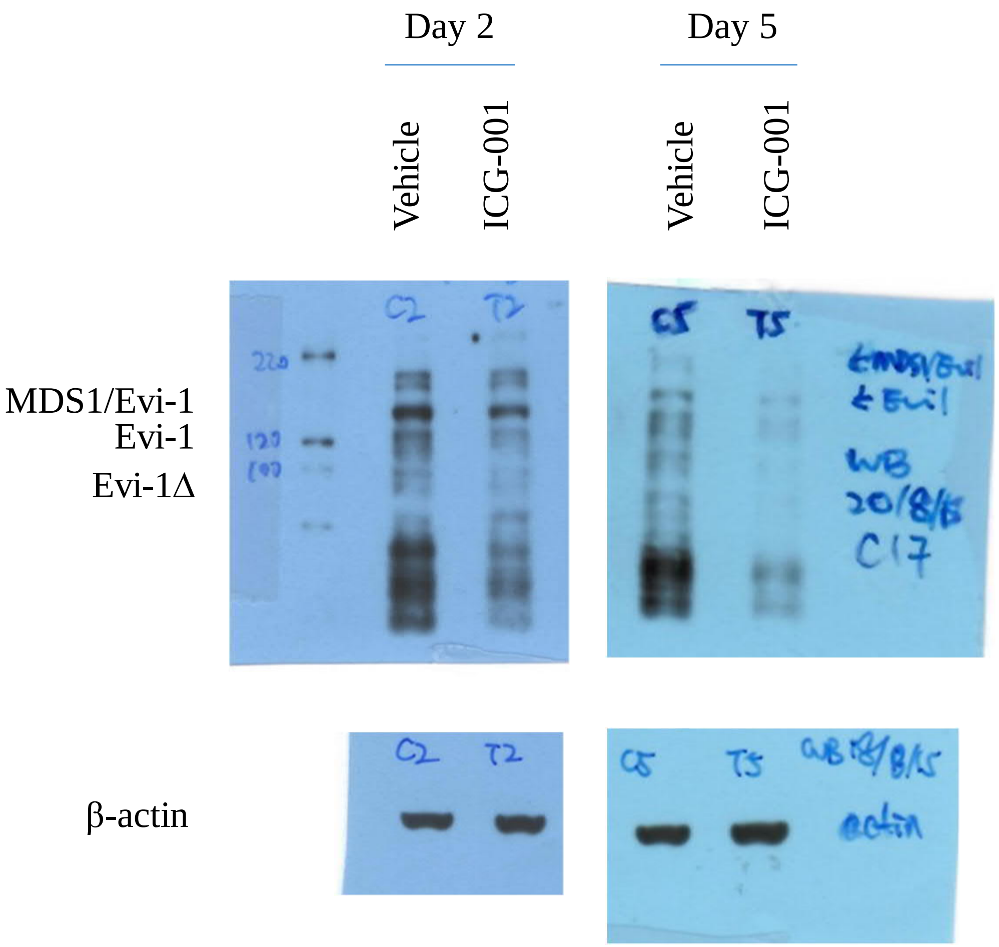

Original western blot image

Figure 2A

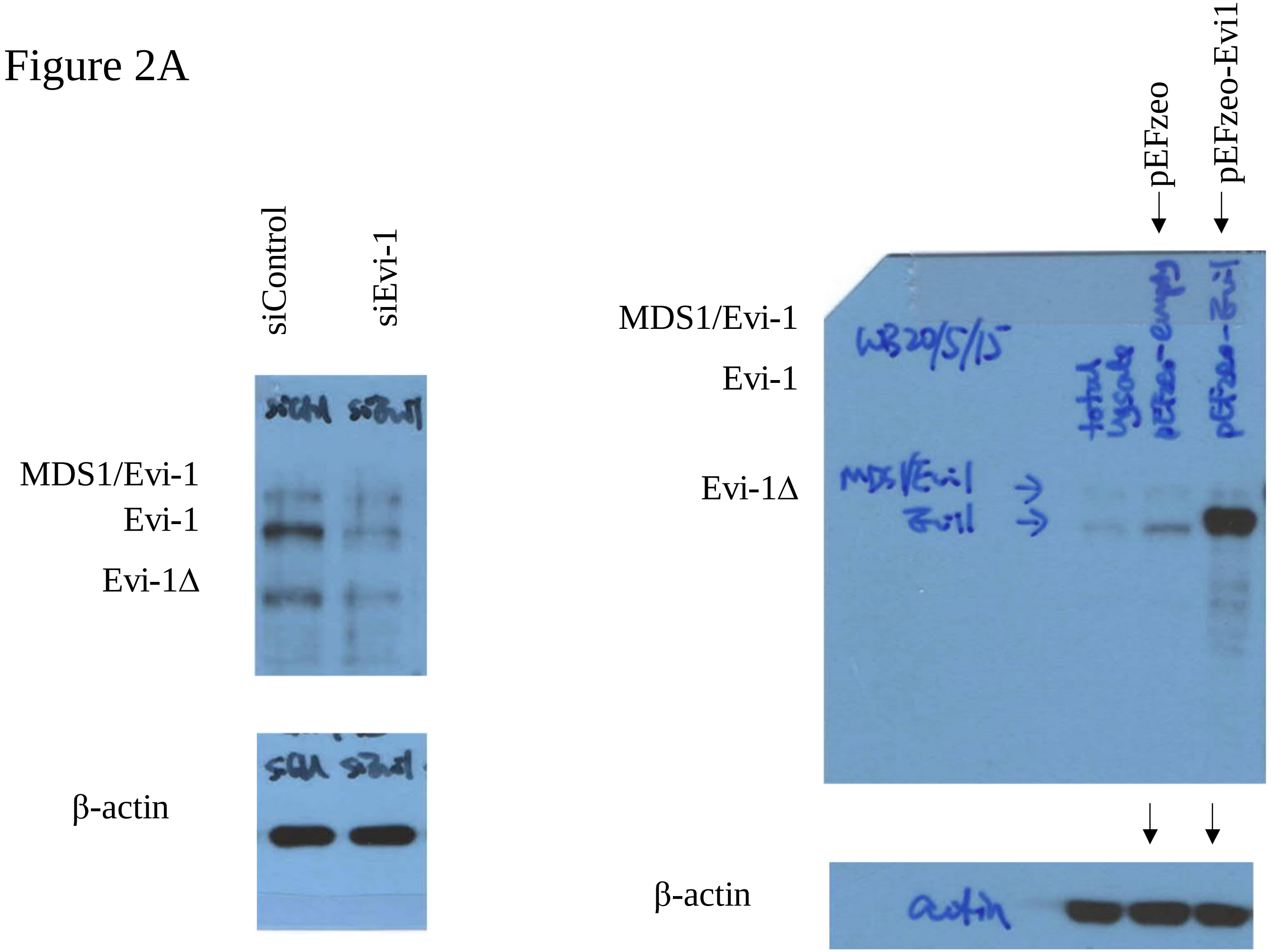

Original western blot image

Figure 3C

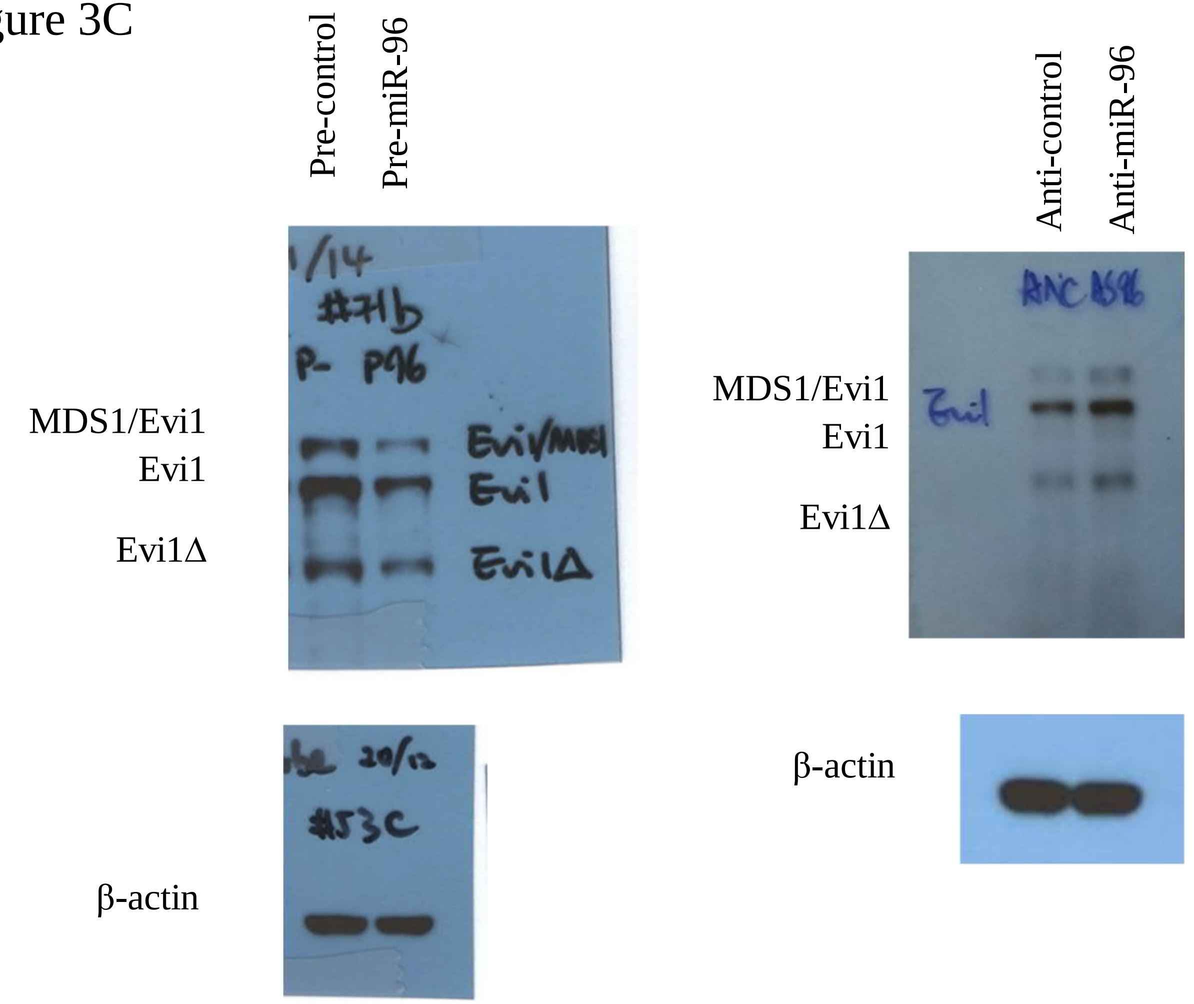

Supplement: Supplementary file 1 [file ijms-21-05495-s001.zip › ijms-833245 -Sup resub ver2/Original WB - 1.pdf]
