# Supplementary figures and images for: Role of miR-96/EVI1/miR-449a Axis in the Nasopharyngeal Carcinoma Cell Migration and Tumor Sphere Formation
Source: Int J Mol Sci. 2020 Jul 31;21(15):5495. doi: 10.3390/ijms21155495 (PMC7432346; doi:10.3390/ijms21155495)

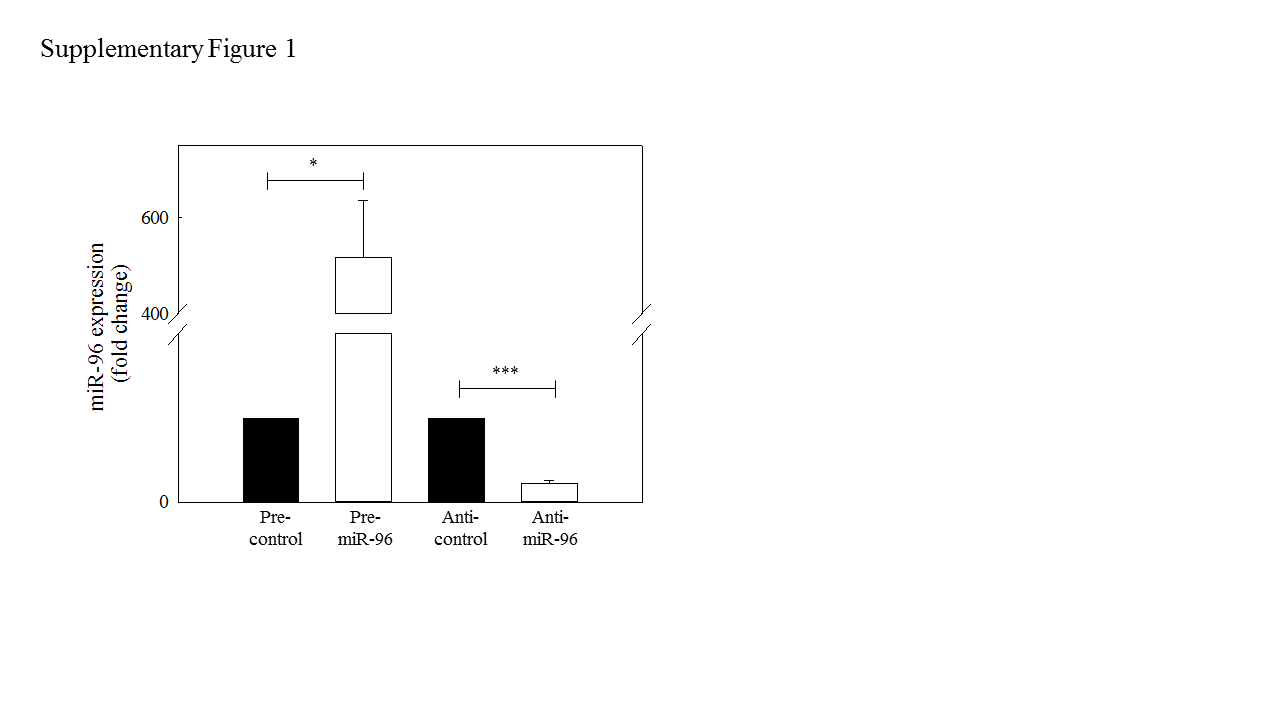

Supplement: Supplementary file 1 [file ijms-21-05495-s001.zip › ijms-833245 -Sup resub ver2/Supplementary Figure 1.TIF]

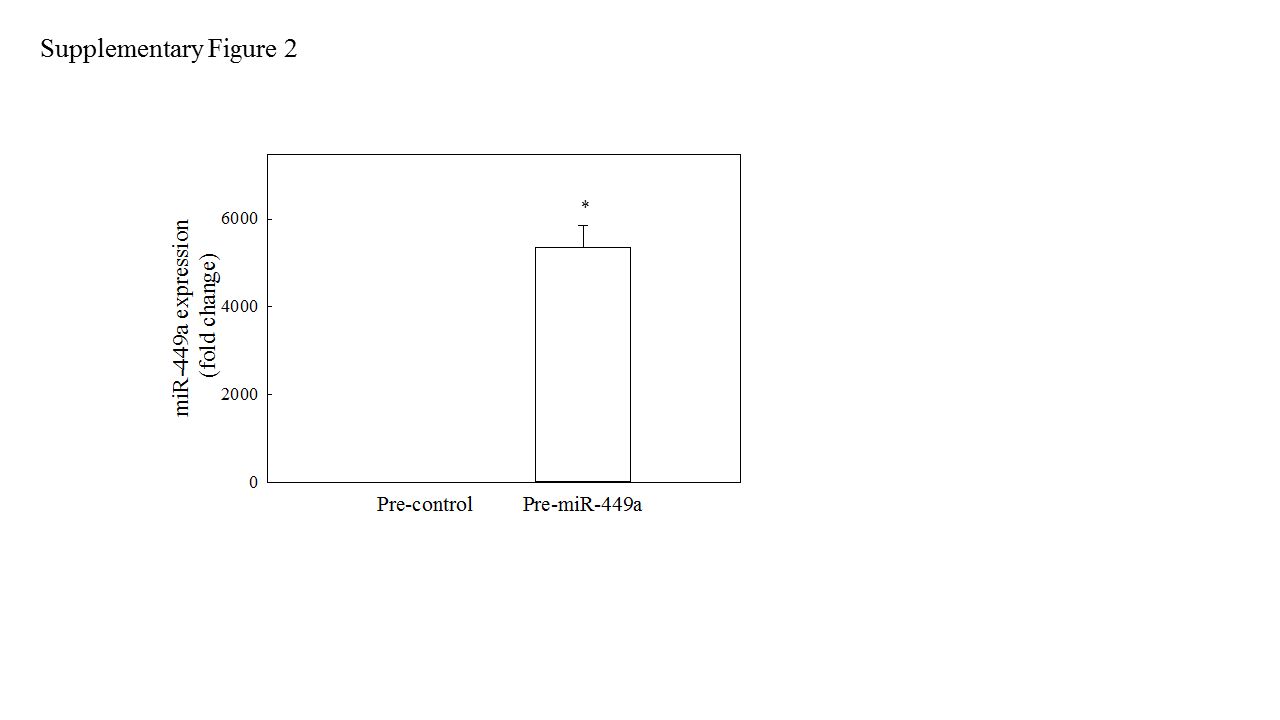

Supplement: Supplementary file 1 [file ijms-21-05495-s001.zip › ijms-833245 -Sup resub ver2/Supplementary Figure 2.TIF]

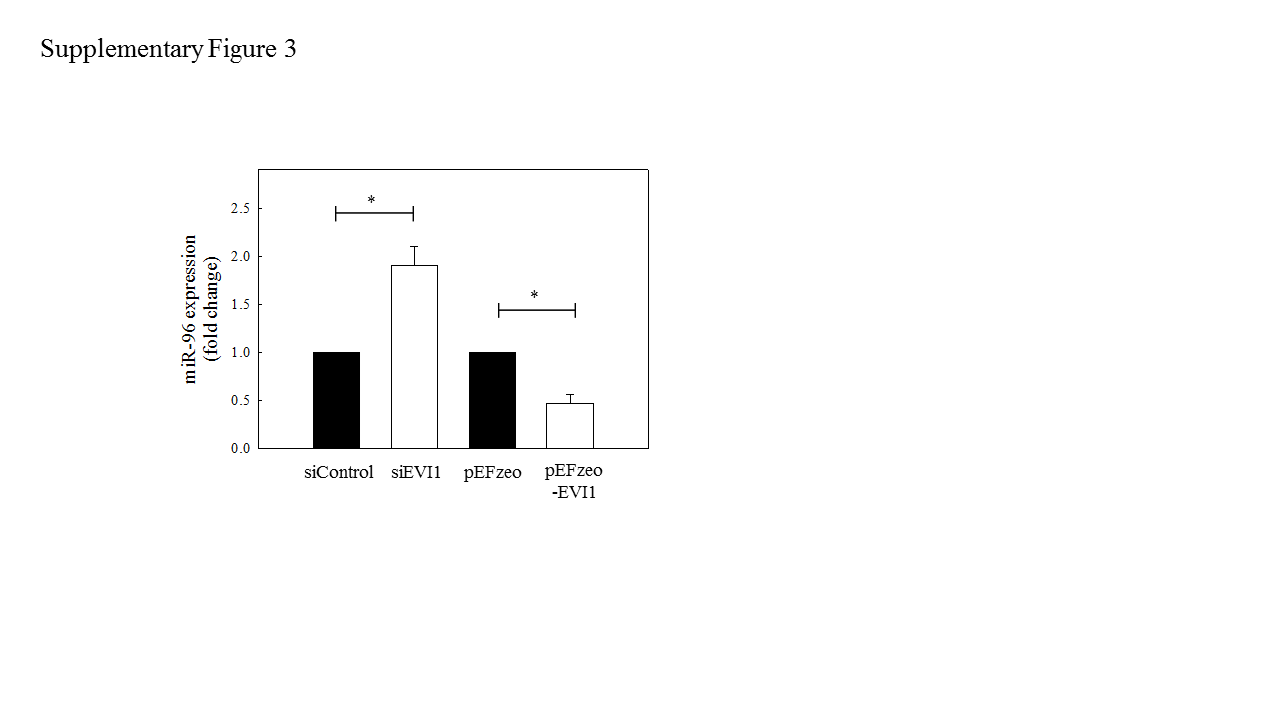

Supplement: Supplementary file 1 [file ijms-21-05495-s001.zip › ijms-833245 -Sup resub ver2/Supplementary Figure 3.TIF]
